# The Respiratory Adjusted Shock Index at Admission Is a Valuable Predictor of In-Hospital Outcomes for Elderly Emergency Patients with Medical Diseases at a Japanese Community General Hospital

**DOI:** 10.3390/jcm13164866

**Published:** 2024-08-18

**Authors:** Taiki Hori, Ken-ichi Aihara, Takeshi Watanabe, Kaori Inaba, Keisuke Inaba, Yousuke Kaneko, Saki Kawata, Keisuke Kawahito, Hiroki Kita, Kazuma Shimizu, Minae Hosoki, Kensuke Mori, Teruyoshi Kageji, Hideyuki Uraoka, Shingen Nakamura

**Affiliations:** 1 Department of Internal Medicine, Tokushima Prefectural Kaifu Hospital, 266 Sugitani, Nakamura, Mugi-cho, Kaifu-gun, Tokushima 775-0006, Japan; 2 Department of Hematology, Endocrinology and Metabolism, Tokushima University Graduate School of Biomedical Sciences, 3-18-15 Kuramoto-cho, Tokushima 770-8503, Japan; 3 Department of Community Medicine and Medical Science, Tokushima University Graduate School of Biomedical Sciences, 3-18-15 Kuramoto-cho, Tokushima 770-8503, Japan; 4 Department of Preventive Medicine, Tokushima University Graduate School of Biomedical Sciences, 3-18-15 Kuramoto-cho, Tokushima 770-8503, Japan; 5 Department of General Medicine, Tokushima University Graduate School of Biomedical Sciences, 3-18-15 Kuramoto-cho, Tokushima 770-8503, Japan; 6 Department of Neurosurgery, Tokushima Prefectural Kaifu Hospital, 266 Sugitani, Nakamura, Mugi-cho, Kaifu-gun, Tokushima 775-0006, Japan; 7 Department of Orthopedic Surgery, Tokushima Prefectural Kaifu Hospital, 266 Sugitani, Nakamura, Mugi-cho, Kaifu-gun, Tokushima 775-0006, Japan

**Keywords:** respiratory adjusted shock index, elderly, emergency room, medical diseases, prognosis

## Abstract

**Background**: The respiratory adjusted shock index (RASI) is a risk score whose usefulness in patients with sepsis and trauma has previously been reported. However, its relevance in elderly emergency patients with medical diseases is yet to be clarified. This study assessed the usefulness of the RASI, which can be evaluated without requiring special equipment, to provide objective and rapid emergency responses. **Methods**: In this retrospective study, we recruited patients with medical diseases, aged 65 years or older, who were transported to the emergency room from Tokushima Prefectural Kaifu Hospital and underwent arterial blood gas testing from 1 January 2022 to 31 December 2023. We investigated the association of the RASI with other indices, including the lactate level, National Early Warning Score 2 (NEWS2), Shock Index (SI), Sequential Organ Failure Assessment (SOFA) score, quick SOFA (qSOFA) score, and systemic inflammatory response syndrome (SIRS). **Results:** In this study, we included 260 patients (mean age, 86 years), of whom 234 were admitted to the hospital; 27 and 49 patients died within 7 and 30 days of admission, respectively. The RASI was positively correlated with the lactate level, NEWS2, SI, and increase in the SOFA score (*p* < 0.001). The RASI was higher in patients with a SIRS or qSOFA score ≥ 2 than in those without (*p* < 0.001). It predicted death within 7 and 30 days of admission with an area under the curve (AUC) of 0.80 (95% confidence interval [CI]: 0.73–0.87), sensitivity of 96.3%, and specificity of 53.6% when the cutoff value was set to 1.58 and with an AUC of 0.73 (95% CI: 0.66–0.81), sensitivity of 69.4%, and specificity of 70.6% when the cutoff value was set to 1.83, respectively. **Conclusions**: The RASI is a simple indicator that can be used for predicting in-hospital outcomes in elderly emergency patients with medical diseases. Larger prospective studies based on this study are needed.

## 1. Introduction

In Japan, many elderly individuals are treated in clinics without hospital beds or visiting doctors. If symptoms worsen, the patient is transported on an emergency basis to a secondary emergency medical facility. Tokushima Prefectural Kaifu Hospital is a general community hospital located in the rural area of Tokushima Prefecture, Japan. Kaifu District is one of the areas where population aging has progressed the most in Japan, and people over 65 years of age account for 50.8% of the population as of 2024 [1]. Kaifu District has approximately 17,000 people and a single emergency hospital. Tokushima Prefectural Kaifu Hospital is a primary and secondary emergency medical facility with approximately 100 beds. The majority of patients in Kaifu District receive initial treatment in this hospital. Since elderly people have a wide variety of illnesses and undergo various treatments, their disease severity is often difficult to ascertain immediately. Japan has the highest proportion of older adults worldwide [2], and the demand for emergency medical care for elderly people is ever increasing. The number of elderly people transported by ambulance is increasing annually, accounting for 62.1% of the total (6.21 million people) population in 2022 [3]. A similar trajectory is expected worldwide soon owing to the increasing proportion of older people. Moreover, since there are areas where medical care resources are limited, a simpler scoring system would be required to assess elderly patients in emergency rooms.

Various clinical laboratory test results and traditional severity risk scores have been used as initial assessments in emergency rooms. Lactate [4,5], the National Early Warning Score 2 (NEWS2) [6,7], the Shock Index (SI) [8], and the systemic inflammatory response syndrome (SIRS) criteria [9,10] can predict the mortality of patients in the emergency room. Lactate levels are measured using a blood gas analyzer and are often impossible to measure in primary medical institutions. The NEWS2 and SI can be evaluated by simply measuring vital signs but are not widely used as screening tools in emergency rooms. The Royal College of Physicians of London proposed the NEWS2 as an indicator to standardize the evaluation and response of acute diseases [11]. It consists of six physiological parameters, namely, (1) respiratory rate (RR), (2) saturation of percutaneous oxygen (SpO_2_), (3) systolic blood pressure (SBP), (4) heart rate (HR), (5) consciousness, and (6) body temperature. The SpO_2_ item score changes depending on the presence or absence of hypercapnic respiratory failure (partial pressure of arterial carbon dioxide (PaCO_2_) > 45 mmHg). Two points are added if oxygen is administered (Figure S1). A medium NEWS2 value (5–6) should prompt an urgent review by a clinician competent in the assessment of acute illness, who should urgently decide whether escalation of care to a team with critical care skills will be required. A high NEWS2 value (7 or more) should prompt emergency assessment, and the patient is usually transferred to a high-dependency care area [12]. The SIRS criteria require evaluation of the white blood cell count, which is not possible immediately after arriving at the hospital.

The Sequential Organ Failure Assessment (SOFA) score [13] and quick SOFA (qSOFA) score [14] are associated with mortality only in patients with suspected infections. Each one is an indicator that includes blood and blood gas tests and requires the evaluation of multiple items. The SOFA score is an index for evaluating severity and prognosis in critically ill patients with organ failure [15]. It was initially targeted at patients with sepsis but was later expanded to include multiple organ failure. It consists of six items, namely, (1) the respiratory system, (2) the coagulation system, (3) liver function, (4) the cardiovascular system, (5) the central nervous system, and (6) the renal system. Each item is given a score of 0 to 4 (Table S1). Sepsis is defined as organ dysfunction caused by infection, and organ dysfunction can be identified as an acute increase in the total SOFA score by 2 or more points according to the third international consensus definition of sepsis and septic shock (Sepsis-3) [16]. An increase in the SOFA score from the usual status was defined as ΔSOFA, as used in the definition of sepsis in Sepsis-3. If the patient’s usual status was unknown, the SOFA score was set to 0. The qSOFA score has been proposed as a tool for general practitioners to screen for sepsis in the initial treatment setting [16]. It comprises three physiological parameters, namely, (1) alteration in mental status, (2) SBP ≤ 100 mmHg, and (3) RR ≥ 22 breaths per min. If ≥2 items on the qSOFA score are positive, sepsis is suspected, and further detailed evaluation should be performed (Sepsis-3).

SIRS has been proposed as an indicator for evaluating non-specific systemic biological reactions caused by immune/inflammatory reactions centered on cytokines, regardless of the type of invasion [17]. It is defined by the satisfaction of any two of the following four criteria: (1) temperature <36 °C or >38 °C, (2) HR >90 beats per min, (3) RR >20 breaths per minute, and (4) white blood cell count: <4000/μL or >12,000/μL or >10% immature band forms.

The respiratory adjusted shock index (RASI) is the SI (HR/SBP) plus an RR component and is calculated according to the formula HR/SBP × RR/10. It has been reported as a simple marker for assessing the presence of occult shock in sepsis [18] and trauma [19]. Several studies have reported that the RASI is associated with mortality in patients with sepsis [20]. Although there are studies in specific areas, such as trauma and sepsis, the applicability of the RASI as a screening tool in elderly emergency patients with medical diseases has not been clarified yet in the context of the emergency room.

In Japan, transportation destinations for emergency patients are being consolidated; however, in community medical care for older adults, decisions regarding transfers to higher-level medical institutions must be made based on evaluations using limited resources. In such settings, indicators that can be evaluated by healthcare workers who are unaccustomed to dealing with emergency patients would be required. Additionally, indicators that do not require blood tests or special equipment could enable prehospital evaluation and prompt action. By demonstrating the usefulness of the RASI in internal medicine, more objective and rapid responses will become possible. Therefore, this study aimed to assess the usefulness of the RASI, which can be evaluated without requiring special equipment, to provide objective and rapid emergency responses.

## 2. Materials and Methods

### 2.1. Study Design, Participants, and Ethics Statement

This was a retrospective study using information from electronic medical records. In this study, patients aged 65 years or older with medical diseases (treated using internal medicine rather than through surgery) who underwent arterial blood gas testing were transported to the Tokushima Prefectural Kaifu Hospital by ambulance between 1 January 2022 and 31 December 2023. Tokushima Prefectural Kaifu Hospital is a community general hospital in the rural area of Tokushima Prefecture. Kaifu district has 18,000 people and only a single emergency hospital. In this study, only patients diagnosed with medical diseases were included. Patients who required surgical treatment but were treated conservatively in consideration of their overall condition were excluded. Patients with out-of-hospital cardiac arrest and those who received pre-hospital treatment other than oxygen administration were ineligible. Blood test results and vital signs were recorded upon arrival at the emergency room, and the RASI, SI, NEWS2, SIRS criteria, SOFA score, and qSOFA score were evaluated. We investigated the association of the RASI with the lactate level, NEWS2, SI, SOFA score, qSOFA score, and SIRS criteria. Next, we compared the RASI between individuals who died or did not die within 7 or 30 days and evaluated the predictive capacity of the RASI and other clinical indices for death within 7 or 30 days.

We further collected information on medications for the treatment of cardiovascular diseases and diabetes that might affect the vital signs of the patients enrolled in this study. This study followed the institutional guidelines of Tokushima Prefectural Kaifu Hospital. The study procedures were conducted in accordance with the Declaration of Helsinki, and the study was approved by the relevant Institutional Review Board (approval number 2023010).

### 2.2. Statistical Analysis

Continuous variables with a normal distribution were expressed as the mean ± standard deviation (SD), and those with a non-normal distribution were expressed as the median (first quartile [Q1]–third quartile [Q3]). We evaluated the association of the RASI with the lactate level, NEWS2, SI, ΔSOFA score, qSOFA score, and SIRS criteria using simple linear regression analysis in GraphPad Prism version 9.4.1 (458) for macOS (GraphPad Software, San Diego, CA, USA). For comparison of RASI values between individuals who died and those who did not die within 7 or 30 days, we performed the Mann–Whitney *U* test using GraphPad Prism 9. The predictive capacity of the RASI and other clinical indices for death within 7 or 30 days was evaluated using the area under the curve (AUC), the Hosmer–Lemeshow test, and decision curve analysis using R (The R Foundation for Statistical Computing, Vienna, Austria) and EZR [21] (Saitama Medical Center, Jichi Medical University, Saitama, Japan), a graphical user interface for R. The cut-off point for each index was estimated based on the Youden index. The predictive capacity of the RASI for death within 7 or 30 days was evaluated and compared in patients treated with and without drugs that were expected to affect SBP and HR. Furthermore, we compared indices including the RASI and the predictive capacities of the RASI for death within 7 or 30 days with and without prehospital oxygen administration using the Mann–Whitney *U* test. The criterion for statistical significance was set at *p* < 0.05.

## 3. Results

### 3.1. Patient Characteristics

Overall, 2229 total ambulance transports occurred over the 2 years. Of these, 1844 (82.7%) patients were aged ≥65 years, and 1172 (52.6%) were patients aged ≥65 years with medical disease. Arterial blood gas tests were performed on the 260 eligible patients included in the study. Table 1 presents the patient characteristics. The mean age of the patients was 86 (range, 81–92) years, and 234 (90.0%) of them were admitted to the hospital; 27 (10.4%) and 49 (18.8%) patients died within 7 and 30 days of admission, respectively. No patient died without being admitted to the hospital. The median value of each index was as follows: RASI 1.62 (1.14–2.18), lactate 1.5 (0.8–2.7) mmol/L, NEWS2 7 (5–10), SI 0.67 (0.55–0.87), and ΔSOFA 3 (1–5). 100 (38.5%) patients had a qSOFA score of ≥2, and 176 (67.7%) patients had SIRS.

### 3.2. Association of RASI with Lactate, NEWS2, SI, ΔSOFA Score, qSOFA Score, and SIRS Criteria

**Figure 1:**
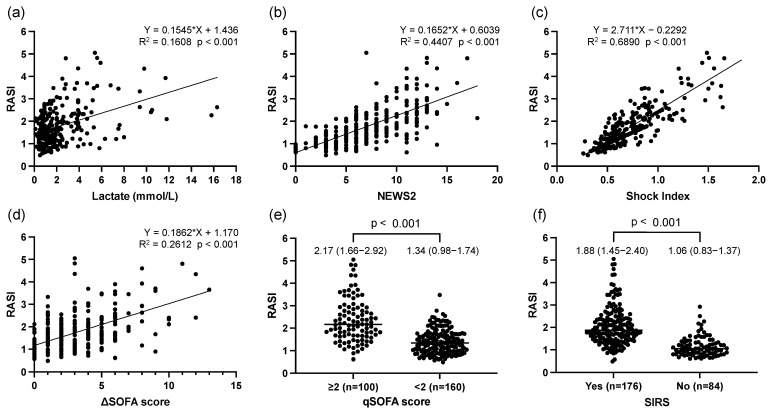
Association of RASI with lactate, NEWS2, Shock Index, ΔSOFA score, and SIRS criteria. (**a**) Association between RASI and lactate. (**b**) Association between RASI and NEWS2. (**c**) Association between RASI and Shock Index. (**d**) Association between RASI and ΔSOFA score. (**e**) Comparison of RASI in patients with qSOFA scores of ≥2 or <2. (**f**) Comparison of RASI in patients with or without SIRS. Abbreviations: RASI: respiratory adjusted shock index; NEWS2: National Early Warning Score 2; SOFA: Sequential Organ Failure Assessment; qSOFA: quick Sequential Organ Failure Assessment; SIRS: systemic inflammatory response syndrome.

The RASI was positively correlated with the lactate level (R^2^ = 0.1608, *p* < 0.001), NEWS2 (R^2^ = 0.4407, *p* < 0.001), SI (R^2^ = 0.6890, *p* < 0.001), and SOFA score (R^2^ = 0.2612, *p* < 0.001) (Figure 1a–d). The RASI was higher in patients with a qSOFA score of ≥2 than in those with a qSOFA score of <2 (2.17 [1.66–2.92] vs. 1.34 [0.98–1.74], *p* < 0.001) (Figure 1e). The RASI was higher in patients with SIRS than in those without (1.88 [1.45–2.40] vs. 1.06 [0.83–1.37], *p* < 0.001) (Figure 1f).

### 3.3. Comparison of RASI between Individuals Who Died within 7 or 30 Days and Those Who Survived

**Figure 2:**
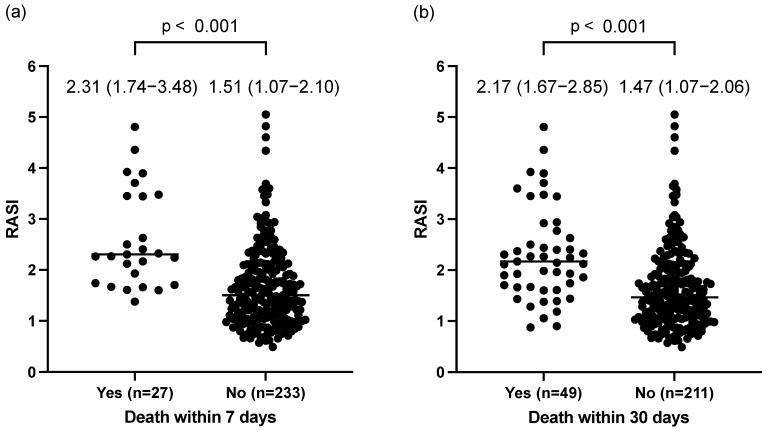
Comparison of RASI between individuals who died and those who did not die within 7 and 30 days. Comparison of RASI in patients who died within 7 (**a**) and 30 days (**b**) after admission. Abbreviation: RASI: respiratory adjusted shock index.

A total of 27 (10.4%) and 49 (18.8%) patients died within 7 and 30 days of admission, respectively. The RASI at admission was higher in patients who died within 7 days (2.31 [1.74–3.48] vs. 1.51 [1.07–2.10], *p* < 0.001) than in those who died within 30 days (2.17 [1.67–2.85] vs. 1.47 [1.07–2.06], *p* < 0.001) (Figure 2a,b).

### 3.4. Predictive Capacity of RASI and Other Clinical Indices for Death within 7 and 30 Days

The predictive capacity of the RASI and other indices for death within 7 days is shown in Table 2. The RASI predicted death within 7 days of admission with an AUC of 0.80 (95% confidence interval [CI]: 0.73–0.87), sensitivity of 96.3%, and specificity of 53.6% when the cutoff value was set to 1.58. It had the highest AUC and sensitivity among all indices. The Hosmer–Lemeshow test showed that the predictions of death within 7 days of admission by all indices, including the RASI, were well calibrated. Only the SIRS score had a significantly lower AUC than the RASI (*p* = 0.028).

The predictive capacity of the RASI and other indices for death within 30 days is shown in Table 3. The RASI predicted death within 30 days of admission with an AUC of 0.73 (95% CI: 0.66–0.81), sensitivity of 69.4%, and specificity of 70.6% when the cutoff value was set to 1.83. Only the AUCs of the RASI, NEWS2, ΔSOFA score, and qSOFA score were higher than 0.70, and they were almost the same for the prediction of death within 30 days. The Hosmer–Lemeshow test showed the predictions for death within 30 days of admission by all indices, including RASI, to be well calibrated. Only the SI had a significantly lower AUC than the RASI (*p* = 0.002).

**Figure 3:**
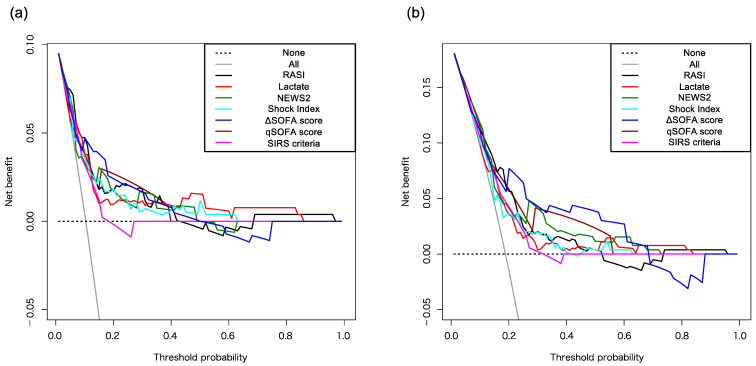
Predictive capacities of RASI, lactate, NEWS2, Shock Index, ΔSOFA score, qSOFA score, and SIRS criteria for death within 7 and 30 days. Predictive capacities for death within 7 days (**a**) and 30 days (**b**) were evaluated using decision curve analysis. Abbreviations: RASI: respiratory adjusted shock index; NEWS2: National Early Warning Score 2; SOFA: Sequential Organ Failure Assessment; qSOFA: quick Sequential Organ Failure Assessment; SIRS: systemic inflammatory response syndrome.

Although the RASI can be quickly and easily evaluated in the emergency room, it can predict death within 7 days with a similar or better predictive capacity than other known indicators in elderly patients with medical diseases (Figure 3a). The RASI was slightly inferior to the NEWS2, ΔSOFA score, and qSOFA score in predicting death within 30 days (Figure 3b). However, these indices showed no significant difference when evaluated using decision curve analysis.

### 3.5. Predictive Capacity of RASI for Death within 7 and 30 Days Compared between Patients on and off Anti-Hypertensive Drugs

Antihypertensive drugs can modify SBP and/or HR, which may, in turn, alter the RASI. Therefore, we investigated whether antihypertensive drug use could affect the predictive capacity of the RASI regarding mortality. The predictive capacity of the RASI for death within 7 or 30 days was compared with and without drugs (Table S2). The AUCs for predicting death within 7 or 30 days was each lower in patients using β-blocker and loop diuretics than in those without. Sixteen (6.2%) patients were taking thiazide, and only one of them died within 30 days of admission. Although the AUC for predicting death within 30 days in patients using thiazide was small, it was an insufficient statistic, since the sample size was quite small.

### 3.6. Comparison of Indices Including the RASI and the Predictive Capacities of the RASI for Mortality within 7 or 30 Days with and without Prehospital Administration

Oxygen administration is expected to affect the respiratory rate. Therefore, we investigated whether prehospital oxygen administration could affect the indices including the RASI and the predictive capacity of the RASI regarding mortality (Table S3). Patients who received prehospital oxygen administration had higher severity indices and higher RASI values than those who did not. The AUC for predicting death within 7 or 30 days was lower in patients who received prehospital oxygen administration than in those who did not.

## 4. Discussion

While the RASI was already known to be evaluated quickly and easily in the emergency room, the current study was the first to conclude that it can be applied to elderly emergency patients with medical diseases.

Elevated lactate levels occur due to tissue hypoperfusion and relative hypoxia due to increased tissue oxygen demand, increased glycolysis, and decreased clearance [22,23]. YJ. Park et al. reported that the AUC of the lactate level was 0.711 (95% CI: 0.703–0.718) for predicting 30-day in-hospital mortality among unselected patients presenting to the emergency department (sensitivity and specificity for the cutoff of >2.6 mmol/L were 56.7% and 74.3%, respectively) [5]. A correlation between lactate level and mortality had previously been observed in elderly emergency patients [24]. The usefulness of the SI and lactate for simultaneously predicting mortality in patients with acute heart failure [25] and multi-trauma has already been reported [26]; however, the correlation between SI and lactate or between the RASI and lactate has not been reported yet. Lactic acidosis is classified as a metabolic acidosis, and patients with acidemia have an increased RR as a compensatory change. The correlation between the RASI and lactate levels can be explained by the increase in lactate levels due to unstable hemodynamics (elevated SI) and the associated increase in RR. The AUC of the RASI was higher for mortality prediction than that of lactate, even though dedicated measurement equipment was not required.

The NEWS2 score includes items, such as RR, SBP, and HR, that are also used in the RASI; therefore, there could be some correlation between the NEWS2 and RASI. In a meta-analysis that included 185,835 patients who visited the emergency room regardless of the disease, AUC, sensitivity, and specificity of NEWS2 for 2-day and 30-day mortality were 0.88 (95% CI: 0.85–0.90), 81%, and 81% vs. 0.80 (95% CI: 0.76–0.83), 76%, and 69%, respectively [7]. In frail older patients (≥75 years old), the AUC of the NEWS2 for 30-day mortality was 0.70 (95% CI: 0.64–0.76), and low accuracy was noted despite statistical significance [27]. the RASI and NEWS2 were more strongly associated with the short-term prognosis of death within 7 days, indicating the higher AUC and sensitivity of the RASI. the NEWS2, which requires more items, has the highest specificity; therefore, it must be used depending on the situation.

The SI and RASI can be expected to be correlated, since the calculation formula is similar for both, and the latter simply adds RR to the SI. The AUC of the RASI (0.614 [95% CI: 0.531–0.697]) predicting mortality during hospitalization has been predicted to be higher than that of SI (0.562 [95% CI: 0.477–0.647]) in patients with sepsis; however, the difference was not statistically significant [20]. In our study, both AUCs were higher than those reported in a previous study, since we investigated short-term prognoses. the RASI has a higher sensitivity than SI, since the vital signs are thought to reflect short-term prognosis.

The SOFA score is used in the intensive care unit to assess disease severity and its correlation with prognosis [28,29]. Since the ΔSOFA score is known as a diagnostic criterion for sepsis in Sepsis-3, many studies have used it on patients with sepsis. The SOFA score cannot be quickly and easily assessed, since it requires blood tests, including gas tests. The positive correlation between SOFA score and RASI, which can be evaluated rapidly, suggested that the RASI could reflect the severity of organ dysfunction. The AUCs of the RASI and ΔSOFA score were equal in predicting death within 7 days, but ΔSOFA score had lower sensitivity. The SOFA score had the highest AUC value for predicting mortality within 30 days. The decision curve analysis also showed it to be superior to the other indicators. It has been reported to be useful in predicting in-hospital mortality in patients with suspected infection [13,30] and in critically ill elderly patients admitted to the intensive care unit [31]. We clarified the usefulness of the RASI rather than the SOFA score for elderly emergency patients with medical diseases in the emergency room.

The qSOFA score was developed as a screening tool to detect suspected sepsis, and the diagnosis of sepsis is made based on the more detailed SOFA score. Therefore, the SOFA score is reportedly a better predictor of prognosis in patients with suspected infection than the qSOFA score [13,14,30,32]. However, the qSOFA and SOFA scores had similar prognostic value in some areas, such as older adults hospitalized with community-acquired urinary tract infections [33]. In this study, the qSOFA score had similar AUC and sensitivity to the SOFA score in predicting 7- or 30-day mortality, suggesting that the qSOFA score is a useful screening tool for medical diseases. Furthermore, the qSOFA score is a simple index similar to the RASI; however, the RASI had a higher AUC and sensitivity for predicting death within 7 days.

The SIRS criteria had previously been used to diagnose sepsis; however, they included cases of low severity and were non-specific. RASI was higher in patients with SIRS than in those without, in this study (1.88 [1.45–2.40] vs. 1.06 [0.83–1.37], *p* < 0.001). The SIRS criteria had a low AUC and sensitivity for predicting prognosis in elderly patients with medical diseases. Decision curve analysis showed that the SIRS criteria were not superior to the other indicators. In previous studies, the AUC of the SIRS criteria for predicting in-hospital death was as low as approximately 0.60 [9,30], and was considered unsuitable as a prognostic indicator.

In elderly patients, SBP and RR increase while HR decreases physiologically, as a result of the progression of arteriosclerosis and organ damage [34]. Elderly people form a heterogeneous group with different background diseases and medications, and their standard values of RR are wider than those of young people (≥65 years old: 12–28 breaths per min) [34]. In order to compensate for stress reduction, the blood pressure, HR, and RR become less responsive to invasion. Therefore, if the RASI increases in elderly patients, the possibility of serious medical conditions should be considered. Since RASI might be able to detect minute changes that are missed by individual evaluations of vital signs, it is practical for use in the fields of geriatric medicine and emergency medicine.

The use of calcium channel or β-blockers has been reported to weaken the association between SI and 30-day mortality [35]. This study included 117 (45.0%) patients using calcium channel blockers and 50 (19.2%) using β-blockers. We investigated the predictive capacity of the RASI for death within 7 or 30 days, based on the presence or absence of drugs that were expected to affect SBP and HR (Table S2). While the use of calcium channel blockers did not reduce the ability of the RASI to predict mortality, β-blockers and loop diuretics slightly decreased the predictive power in this study. β-blockers suppressed the increase in HR caused by stress response and masked the symptoms of hypoglycemia and hypotension [36]. Although patients using β-blockers should be careful of under-triage, this study, which included 19.2% of patients using β-blockers overall, showed the usefulness of RASI. Among the patients using loop diuretics for fluid management, 28 (34.1%) were additionally taking β-blockers to control blood pressure and HR or to protect the heart. This was higher than the proportion of β-blockers used in patients not using loop diuretics (12.4%), hence suggesting that β-blockers may have been a confounding factor contributing to the decline in the predictive capacity of RASI for mortality within 7 or 30 days.

Oxygen administration corrects hypoxia and reduces the increase in respiratory rate as a response to hypoxia. The need for oxygen administration, whether due to hypoxia or an unmeasurable condition caused by reduced peripheral blood flow, is a serious condition; however, the decrease in respiratory rate is believed to lower the RASI. This may affect the prediction of short-term prognosis using the RASI. The AUC for predicting death within 7 or 30 days was lower in patients who received prehospital oxygen administration than in those who did not. However, considering the current state of the emergency departments, indicators that cannot be used for patients receiving prehospital oxygen administration are of minimal value; therefore, this study included patients receiving prehospital oxygen administration. In future research, considering the assessment of the RASI before oxygen administration is necessary.

This study had some limitations. We included only patients who underwent arterial blood gas tests at the discretion of the attending physician because we wanted to evaluate the relationship between the RASI and traditional prognostic indicators, such as lactate and the SOFA score. Arterial blood gas tests were not performed in all patients transported to the emergency room. They were more likely to be performed when vital signs were abnormal, particularly oxygenation. Therefore, we may not have included patients whose symptoms appeared to be mild at the time of transportation, but whose symptoms subsequently worsened. Considering the real-world situation in the emergency department, we included patients who had been started on oxygen therapy before arrival. Predictive capacities may be poorer in patients receiving prehospital oxygen administration; therefore, studies including assessment before oxygen therapy are needed. Since the AUC of the RASI for predicting prognosis was not statistically superior compared to that of other indices, the use of the RASI alone was not recommended; rather, it should be used depending on the situation. This study only evaluated the RASI as an initial assessment at the time of transportation. Vital signs change over time, and changes in the RASI due to medical treatment might predict prognosis more strongly; however, this could not be verified in this study. Furthermore, whether the RASI can be applied to patients in hospitals and those with a wider range of diseases than just medical diseases would need to be examined in future studies.

## 5. Conclusions

The RASI enables quick and easy assessment and may be more advantageous than other traditional severity risk scores for evaluating disease severity and predicting short-term prognosis in elderly emergency patients with medical diseases.

This study was retrospective and conducted in one of the areas where population aging has progressed the most in Japan. Since this was a single-center study, further prospective studies based on this study are needed to investigate whether it can be adapted for the elderly emergency patients in other districts.

## Figures and Tables

**Table 1:** Patient characteristics.

	Total Subjects (n = 260)
Age (years)	86 (81–92)
Male (n, (%))	126 (48.5)
Admission (n, (%))	234 (90.0)
Prehospital oxygen administration (n, (%))	160 (61.5)
Death within 7 days of admission (n, (%))	27 (10.4)
Death within 30 days of admission (n, (%))	49 (18.8)
Diseases that led to emergency transport (n, (%))	
Diseases of the respiratory system	98 (37.7)
Diseases of the circulatory system	64 (24.6)
Diseases of the digestive system	27 (10.4)
Endocrine, nutritional, or metabolic diseases	20 (7.7)
Diseases of the genitourinary system	19 (7.3)
Certain infectious or parasitic diseases	12 (4.6)
Neoplasms	9 (3.5)
Diseases of the skin	4 (1.5)
Diseases of the musculoskeletal system or connective tissue	4 (1.5)
Others	3 (1.2)
Comorbidities (n, (%))	
Hypertension	179 (68.8)
Diabetes mellitus	61 (23.5)
Dyslipidemia	50 (19.2)
Medications used (n, (%))	
ARB/ACEi	90 (34.6)
Calcium channel blocker	117 (45.0)
β-Blocker	50 (19.2)
Loop diuretic	82 (31.5)
Thiazide	16 (6.2)
Mineralocorticoid receptor antagonist	33 (12.7)
Initial evaluation of vital signs	
Glasgow Coma Scale (points)	14 (13–15)
Systolic blood pressure (mmHg)	132 (111–153)
Diastolic blood pressure (mmHg)	77 (65–90)
Mean blood pressure (mmHg)	96 (80–110)
Heart rate (/min)	92 (75–108)
Respiratory rate (/min)	23 (20–28)
Percutaneous oxygen saturation (%)	97 (93–98)
Body temperature (°C)	37.0 (36.4–37.9)
National Early Warning Score 2 (NEWS2)	7 (5–10)
Shock Index (SI)	0.67 (0.55–0.87)
Respiratory adjusted shock index (RASI)	1.62 (1.14–2.18)
Quick Sequential Organ Failure Assessment (qSOFA) score ≥ 2 (n, (%))	100 (38.5)
Initial arterial blood gas tests	
pH	7.44 (7.38–7.48)
PaCO_2_ (mmHg)	33.0 (28.3–39.8)
PaO_2_ (mmHg)	85 (67–109)
*p*/F ratio	316 (204–435)
HCO_3_^−^ (mmol/L)	22.4 (19.3–26.0)
Base excess (mEq/L)	−0.9 (−4.3–1.8)
Lactate (mmol/L)	1.5 (0.8–2.7)
Initial peripheral blood tests	
White blood cells (×10^3^/μL)	8.25 (5.76–11.53)
Hemoglobin (g/dL)	10.9 (9.6–12.7)
Platelets (×10^3^/μL)	18.6 (14.0–23.6)
Total bilirubin (mg/dL)	0.7 (0.6–1.0)
Creatinine (mg/dL)	1.03 (0.76–1.47)
Systemic inflammatory response syndrome (SIRS) (n, (%))	176 (67.7)
ΔSequential Organ Failure Assessment (SOFA) score	3 (1–5)

Abbreviations: ARB: angiotensin II receptor blocker; ACEi: angiotensin-converting enzyme inhibitor; pH: potential hydrogen; PaCO_2_: partial pressure of arterial carbon dioxide; PaO_2_: partial pressure of arterial oxygen; *p*/F: partial pressure of arterial oxygen/fraction of inspiratory oxygen; HCO_3_^−^: bicarbonate.

**Table 2:** The predictive capacity of the RASI and other clinical indices for death within 7 days.

Death within 7 Days						
	Cutoff	AUC (95%CI)	Sensitivity (%)	Specificity (%)	Hosmer-Lemeshow Test	*p* Value (vs. RASI)
RASI	1.58	0.80 (0.73–0.87)	96.3	53.6	*p* = 0.10	−
Lactate	2.20	0.73 (0.62–0.84)	70.4	72.5	*p* = 0.33	0.230
NEWS2	10	0.76 (0.67–0.84)	55.6	82.8	*p* = 0.08	0.221
Shock Index	0.75	0.73 (0.62–0.83)	74.1	62.7	*p* = 0.53	0.055
ΔSOFA score	4	0.78 (0.68–0.87)	66.7	77.7	*p* = 0.80	0.638
qSOFA score	2	0.75 (0.66–0.84)	70.4	65.2	*p* = 0.56	0.308
SIRS score	2	0.69 (0.60–0.78)	63	66.1	*p* = 0.86	0.028

Abbreviations: AUC: area under the curve; CI: confidence interval; RASI: respiratory adjusted shock index; NEWS2: National Early Warning Score 2; ΔSOFA: delta Sequential Organ Failure Assessment; qSOFA: quick Sequential Organ Failure Assessment; SIRS: systemic inflammatory response syndrome.

**Table 3:** The predictive capacity of the RASI and other clinical indices for death within 30 days.

Death within 30 Days						
	Cutoff	AUC (95%CI)	Sensitivity (%)	Specificity (%)	Hosmer-Lemeshow Test	*p* Value (vs. RASI)
RASI	1.83	0.73 (0.66–0.81)	69.4	70.6	*p* = 0.68	−
Lactate	1.60	0.68 (0.60–0.76)	69.4	62.1	*p* = 0.37	0.231
NEWS2	10	0.72 (0.65–0.80)	49	85.3	*p* = 0.23	0.698
Shock Index	0.92	0.65 (0.56–0.74)	38.8	85.3	*p* = 0.81	0.002
ΔSOFA score	4	0.74 (0.66–0.82)	61.2	81	*p* = 0.49	0.872
qSOFA score	2	0.73 (0.66–0.80)	65.3	67.8	*p* = 0.58	0.860
SIRS score	2	0.66 (0.58–0.74)	57.1	67.8	*p* = 0.98	0.081

Abbreviations: AUC: area under the curve; CI: confidence interval; RASI: respiratory adjusted shock index; NEWS2: National Early Warning Score 2; ΔSOFA: delta Sequential Organ Failure Assessment; qSOFA: quick Sequential Organ Failure Assessment; SIRS: systemic inflammatory response syndrome.

## Data Availability

Data will be made available on reasonable request.

## Supplementary Materials

The following supporting information can be downloaded at: https://www.mdpi.com/article/10.3390/jcm13164866/s1. Figure S1: Description of National Early Warning Score 2 (NEWS2). The SpO_2_ scoring scale (Scale 2) on the chart should be used for patients confirmed to have prior or current hypercapnic respiratory failure. The total score for all items can range from 0 to 20 points. Abbreviations: SpO_2_: percutaneous oxygen saturation; Table S1: Description of Sequential Organ Failure Assessment (SOFA) score; Table S2: The predictive capacities of RASI for death within 7 or 30 days compared between cases with and without drugs; Table S3: Comparison of indices including RASI and the predictive capacities of RASI for death within 7 or 30 days with and without prehospital administration.
