# Supplementary material for: The Respiratory Adjusted Shock Index at Admission Is a Valuable Predictor of In-Hospital Outcomes for Elderly Emergency Patients with Medical Diseases at a Japanese Community General Hospital
Source: J Clin Med. 2024 Aug 18;13(16):4866. doi: 10.3390/jcm13164866 (PMC11355747; doi:10.3390/jcm13164866)
Supplement: Supplementary file 1 [file jcm-13-04866-s001.zip › jcm-3087780-supplementary.pdf]

Table S1. Description of Sequential Organ Failure Assessment (SOFA) score

System	Score				
	0	1	2	3	4
Respiration<br>PaO_2/FiO_2	≥400	<400	<300	<200<br>with respiratory support	<100<br>with respiratory support
Coagulation<br>Platelets (×10^3/μL)	≥150	<150	<100	<50	<20
Liver<br>Bilirubin (mg/dL)	<1.2	1.2-1.9	2.0-5.9	6.0-11.9	>12.0
Cardiovascular	MAP ≥70mmHg	MAP <70mmHg	DOA <5γ or<br>DOB (any dose)	DOA 5.1-15γ<br>or Ad ≤0.1γ<br>or NOA ≤0.1γ	DOA >15γ<br>or Ad >0.1γ<br>or NOA >0.1γ
Central nervous system<br>Glasgow Coma Scale	15	13-14	10-12	6-9	<6
Renal<br>Creatinine (mg/dL)<br>Urine output (mL/day)	<1.2	1.2-1.9	2.0-3.4	3.5-4.9<br><500	>5.0<br><200

Abbreviations: PaO_2: partial pressure of arterial oxygen; FiO_2: fraction of inspiratory oxygen; MAP: mean arterial pressure; DOA: dopamine; DOB: dobutamine; Ad: adrenalin; NOA: noradrenalin

Table S2. The predictive capacities of RASI for death within 7 or 30 days compared between with and without drugs

	Total subjects	Death within 7 days		Death within 30 days	
	(n=260)	n (%)	AUC of RASI (95% CI)	n (%)	AUC of RASI (95% CI)
ARB/ACEi					
Yes	90 (32.1)	4 (4.4)	0.89 (0.75-1.00)	12 (13.3)	0.85 (0.76-0.94)
No	190 (73.1)	23 (12.1)	0.76 (0.67-0.85)	37 (19.5)	0.68 (0.58-0.77)
Calcium channel blocker					
Yes	117 (45.0)	8 (6.8)	0.86 (0.73-0.99)	16 (13.7)	0.78 (0.65-0.92)
No	143 (55.0)	19 (13.3)	0.75 (0.65-0.85)	33 (23.1)	0.69 (0.59-0.78)
β blocker					
Yes	50 (19.2)	6 (12.0)	0.75 (0.57-0.93)	9 (18.0)	0.66 (0.45-0.87)
No	210 (80.8)	21 (10.0)	0.81 (0.73-0.89)	40 (19.0)	0.75 (0.67-0.83)
Loop diuretics					
Yes	82 (31.5)	7 (8.5)	0.76 (0.57-0.95)	16 (19.5)	0.72 (0.58-0.86)
No	178 (68.5)	20 (11.2)	0.82 (0.75-0.90)	33 (18.5)	0.74 (0.65-0.83)
Thiazide					
Yes	16 (6.2)	0 (0.0)	NA	1 (6.3)	0.60 (NA-NA)
No	244 (93.8)	27 (11.1)	0.80 (0.72-0.87)	48 (19.7)	0.74 (0.66-0.81)
Mineralocorticoid receptor antagonist					
Yes	33 (12.7)	3 (9.1)	0.81 (0.53-1.00)	7 (21.1)	0.81 (0.67-0.96)
No	227 (87.3)	24 (10.6)	0.80 (0.72-0.88)	42 (18.5)	0.72 (0.63-0.80)

Abbreviations: ARB: angiotensin II receptor blocker; ACEi: angiotensin-converting enzyme inhibitor; AUC: area under the curve; CI: confidence interval; NA: not available

Table S3. Comparison of indices including RASI and the predictive capacities of RASI for death within 7 or 30 days with and without prehospital oxygen administration

	Prehospital oxygen administration		
	Yes	No	p value
Total subjects (n, (%))	160 (61.5)	100 (38.5)	—
Admission (n, (%))	151 (94.4)	83 (83.0)	0.003
National Early Warning Score 2 (NEWS2)	6 (2-9)	5 (3-7)	<0.001
Shock Index (SI)	0.74 (0.58-0.89)	0.61 (0.49-0.80)	0.004
Respiratory Adjusted Shock Index (RASI)	1.75 (1.36-2.37)	1.26 (0.90-1.82)	<0.001
Systemic Inflammatory Response Syndrome (SIRS) (n, (%))	121 (75.6)	55 (55.0)	0.001
ΔSequential Organ Failure Assessment (SOFA) score	4 (2-5)	2 (1-3)	<0.001
Quick Sequential Organ Failure Assessment (qSOFA) score ≥ 2 (n, (%))	71 (44.4)	29 (29.0)	0.013
Death within 7 days			
n (%)	21 (13.1)	6 (6.0)	0.067
AUC of RASI (95% CI)	0.74 (0.63-0.84)	0.90 (0.81-1.00)	—
Death within 30 days			
n (%)	37 (23.1)	12 (12.0)	0.026
AUC of RASI (95% CI)	0.69 (0.60-0.79)	0.78 (0.65-0.91)	—

Abbreviations: AUC: area under the curve; CI: confidence interval

Chart 1: The NEWS scoring system

Physiological parameter	Score						
	3	2	1	0	1	2	3
Respiration rate (per minute)	≤8		9–11	12–20		21–24	≥25
SpO_2 Scale 1 (%)	≤91	92–93	94–95	≥96			
SpO_2 Scale 2 (%)	≤83	84–85	86–87	88–92<br>≥93 on air	93–94 on oxygen	95–96 on oxygen	≥97 on oxygen
Air or oxygen?		Oxygen		Air			
Systolic blood pressure (mmHg)	≤90	91–100	101–110	111–219			≥220
Pulse (per minute)	≤40		41–50	51–90	91–110	111–130	≥131
Consciousness				Alert			CVPU
Temperature (°C)	≤35.0		35.1–36.0	36.1–38.0	38.1–39.0	≥39.1	

Royal College of Physicians. *National Early Warning Score (NEWS) 2: Standardising the assessment of acute-illness severity in the NHS.* Updated report of a working party. London: RCP, 2017.

Supplementary Figure S1: Description of National Early Warning Score 2 (NEWS2). The SpO_2 scoring scale (Scale 2) on the chart should be used for patients confirmed to have prior or current hypercapnic respiratory failure. The total score for all items can range from 0 to 20 points. Abbreviations: SpO_2: percutaneous oxygen saturation
